# Clinical outcome of rapid diagnosis and antibiotic stewardship in patients with bloodstream infection in Lampang Hospital

**DOI:** 10.1017/ash.2022.311

**Published:** 2022-10-28

**Authors:** Parichart Sakulkonkij, Jackrapong Bruminhent

**Affiliations:** 1 Division of Infectious Diseases, Internal Medicine Department, Lampang Hospital, Lampang, Thailand; 2 Division of Infectious Diseases, Department of Medicine, Faculty of Medicine Ramathibodi Hospital, Mahidol University, Bangkok, Thailand

## Abstract

Bloodstream infection is a significant cause of morbidity and mortality. Early diagnosis and appropriate antibiotic treatment contribute to a favorable prognosis. We demonstrate a reduction of time to proper antibiotics and reduced mortality utilizing prompt diagnosis and antibiotic stewardship by infectious diseases physicians at a general hospital in Thailand.

Early administration of optimal antibiotics is considered the cornerstone therapy for managing severe sepsis and septic shock.^1,2^ Furthermore, previous studies have reported survival benefits of earlier antibiotic administration.^3,4^ Therefore, we assessed time to appropriate antibiotic therapy, clinical outcome, and microbiological outcome after rapid diagnosis was implemented in combination with antibiotic stewardship.

## Determining time to appropriate antibiotic

We obtained data from the internal medicine ward at Lampang Hospital. The medical records of 403 patients were included from June 1, 2019, to December 31, 2019 (ie, the preintervention period) were retrospectively reviewed with informed consent waived. For the postintervention period (from June 1, 2020, to December 31, 2020), the medical records of 309 patients were reviewed with informed consent. All patients were aged ≥18 years and had clinically significant bloodstream infections (BSIs). We applied the following exclusion criteria : contaminated positive blood cultures, polymicrobial bacteremia> 2 species, and/or palliative care. The study protocol was approved by Lampang Hospital Ethics Committees (code no. 119/62).

## Microbiology methods and statistical analyses

Laboratory techniques included MicroScan Walk Away DxM 1096 before rapid diagnosis was implemented in combination with antibiotic stewardship and Phoenix M50 thereafter. Antimicrobial susceptibility testing (AST) was performed using CLSI 2019–2020.^5^ The medical technician enrolled postintervention candidates for 8 hours daily (8:00 a.m.–4:00 p.m.) and notified the ID physician when needed. Empirical antibiotics were adjusted based on clinical syndrome and antibiotic adjustments according to AST results were considered by the ID physician.

We compared the median time to appropriate antibiotics before and after the intervention. Overall survival was assessed using Kaplan-Meier survival analysis. Statistical significance was defined as a *P* < .05. Statistical analysis was carried out using Stata version 14.2 software (StataCorp, College Station, TX).

We included 403 patients before the intervention and 309 patients after the intervention. Community-acquired infection was the most common infection type (*P* = .03). Frequent comorbidities were diabetes mellitus, chronic renal failure, and malignancy. Secondary BSIs were present in 73% of patients before the intervention and 80% of the intervention. Common sites of infection were genitourinary tract, intra-abdominal tract, and respiratory tract. Septic shock (*P* = .03), Pitt bacteremia score (*P* = .007), and SOFA score (*P* = .006) were significantly higher in the preintervention group (Table 1). Gram-negative bacilli were isolated in 64% of cultures before the intervention and 68% of cultures after the intervention. Enterobacterales were the most common pathogens. Before and after the intervention, respectively, most pathogens were antibiotic-susceptible strains (85.6% vs 79.9%), followed by multidrug-resistant (MDR) strains (12.7% vs 15.5%) and extensively drug-resistant (XDR) strains (1.7% vs 4.5%; *P* = .04).

**Table 1.** Characteristics of Patients with Bloodstream Infection

Characteristic	Pre-intervention (N = 403)	Post-intervention (N = 309)	*p*-value
Age, median (IQR) – yr	65 (54–76)	66 (58–76)	0.286
Male sex – no (%)	249 (61.79)	185 (59.87)	0.603
Comorbidity – no (%)			
Malignancy	67 (16.63)	75 (24.27)	0.011
Diabetes mellitus	128 (31.76)	105 (33.98)	0.532
Chronic kidney disease	97 (24.07)	58 (18.77)	0.089
Severity of Illness			
Pitt Bacteremia Score, median (IQR)	2 (0–4)	1 (0–3)	0.007
SOFA Score, median (IQR)	4 (2–8)	4 (2–7)	0.006
Septic Shock - no (%)	112 (27.79)	64 (20.71)	0.030
Site of Acquisition - no (%)			0.034
Community acquired	237 (58.81)	159 (51.46)	
Healthcare-associated	122 (30.27)	97 (31.39)	
Hospital acquired	44 (10.92)	53 (17.15)	
Source of Bloodstream Infection- no (%)			
Primary bloodstream infection	108 (26.80)	62 (20.06)	0.037
Respiratory tract infection	51 (12.66)	22 (7.12)	0.016
Genitourinary tract infection	109 (27.05)	78 (25.24)	0.588
Intraabdominal infection	36 (8.93)	46 (14.89)	0.014
Microorganism			
* E. coli*	124 (30.76)	88 (28.48)	0.508
* Streptococcus* Spp.	82 (20.35)	58 (18.77)	0.600
* S. aureus*	50 (12.41)	32 (10.36)	0.396
* K. pneumonia*	45 (11.17)	31 (10.03)	0.627

IQR, interquartile range.

## Primary and secondary outcomes

**Fig. 1.**
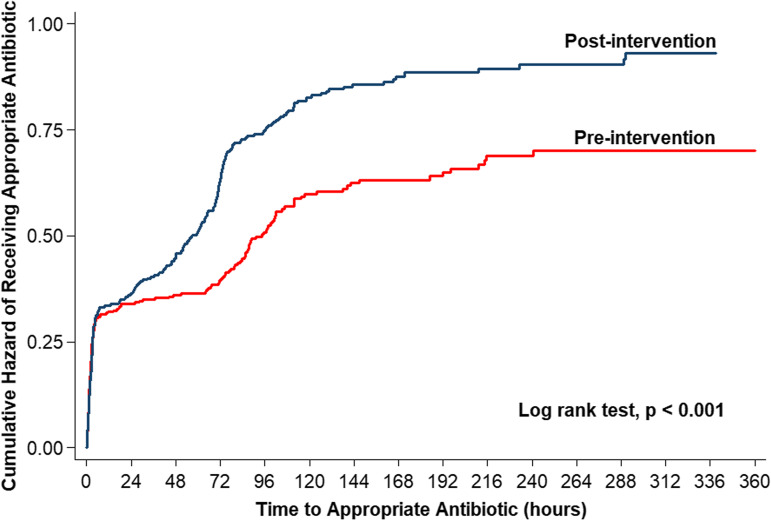
Cumulative hazard of Receiving Appropriate Antibiotic using Kaplan-Meier Analysis.

Overall appropriate antibiotic increased after the intervention (49% vs 80%; *P* < .001), and de-escalation increased after the intervention (13% vs 35%; *P* < .001). Time to appropriate antibiotic decreased from 106.6 hours before the intervention to 61.8 hours after the intervention (*P* < .001) (Fig. 1). Also, the rate of microbiological cure improved after the intervention (53% vs 86%; *P* < .001), and 14-day mortality decreased after the intervention (31% vs 17%; *P* < .001). However, recurrent bacteremia in 1 month remained the same rate (11% vs 13%). Before and after the intervention, respectively, the most common appropriate antibiotics were ceftriaxone (33% vs 43%), followed by ceftazidime (4% vs 6%) and cloxacillin (2% vs 7%).

## Discussion

Our results highlight the importance of implementing a rapid diagnosis combination with antibiotic stewardship to improve outcomes for patients with BSI. We demonstrated that community-acquired BSI and gram-negative bacteria, especially Enterobacterales, were the most common causes of BSI. Patients in the preintervention group had a higher rate of septic shock, higher Pitt bacteremia scores, and higher SOFA scores, so they had a greater likelihood of an unfavorable clinical response.^6^ We documented earlier times to appropriate antibiotic therapy with higher antibiotic adjustment, especially de-escalation in the postintervention group. Rapid diagnosis and administration of appropriate antibiotic therapy and earlier AST-based de-escalation increased the survival rate.^7^ Our findings strongly support using β-lactam monotherapy due to mainly community-acquired BSI with antibiotic-susceptible pathogens because there is no survival benefit from empirical combination therapy over monotherapy in patients with community-acquired, gram-negative BSIs.^8^ Although microbiological cure significantly increased, there was no difference in the proportion of recurrent bacteremia cases within 30 days between the pre- and postintervention groups. Therefore, repeated blood culture may have little utility in those who demonstrate early favorable clinical response.^9^ Finally, our clinical outcomes in BSIs were improved by emphasizing the importance of combining rapid diagnosis with antibiotic stewardship.

This study had several limitations. The quasi-experimental design limited data accessibility. Second, the variation of time to enrollment affects time to antibiotic adjustment. Third, it is difficult to determine appropriate duration of antibiotic, conversion to an oral antibiotic, and source control measures. Lastly, cultures from nonsterile sites are challenging to interpret.

In conclusion, antibiotic stewardship combined with rapid diagnosis contributes to appropriate early antibiotic therapy and enhances recovery.
